# Retrovirus infected cells contain viral microRNAs

**DOI:** 10.1186/1742-4690-10-15

**Published:** 2013-02-07

**Authors:** Zachary A Klase, Gavin C Sampey, Fatah Kashanchi

**Affiliations:** 1 Molecular Virology Section, Laboratory of Molecular Microbiology, National Institute of Allergy and Infectious Diseases, 9000 Rockville Pike, Bethesda, MD 20810, USA; 2 National Center for Biodefense and Infectious Disease, School of Systems Biology, George Mason University, 10900 University Blvd, Manassas, VA 20108, USA

**Keywords:** Retrovirus, microRNA, Bovine leukemia virus, Human immunodeficiency virus, West nile virus, Transcriptional gene silencing, RNA polymerase III

## Abstract

The encoding of microRNAs in retroviral genomes has remained a controversial hypothesis despite significant supporting evidence in recent years. A recent publication demonstrating the production of functional miRNAs from the retrovirus bovine leukemia virus adds further credence to the fact that retroviruses do indeed encode their own miRNAs. Here we comment on the importance of this paper to the field, as well as examine the other known examples of miRNAs encoded by RNA viruses.

## Background

The importance of microRNAs (miRNAs) and other small non-coding RNAs (sncRNAs) in human disease is becoming increasingly evident. In viral infections of humans, the outcome of infection can be influenced by miRNAs encoded by both the cell and the virus [1]. For DNA viruses, several hundred viral miRNAs have been described; however, the description of miRNAs derived from RNA viruses is relatively rare. A paper recently published in the Proceedings of the National Academy of Sciences by Kincaid *et al.* describes several miRNAs encoded by the retrovirus bovine leukemia virus (BLV). The authors demonstrate that these miRNAs are encoded on RNA polymerase III (Pol III) transcripts and identify one that mimics a cellular oncogenic miRNA [2]. While the Pol III transcription of miRNAs has been identified previously in DNA viruses and higher organisms [3], this is the first description of Pol III based miRNA transcription from a RNA virus. In this work, the authors failed to identified Pol III transcribed miRNAs from the five retroviruses of the subfamily *Spumaretrovirinae* they examined but did detect numerous miRNAs from the one *Orthoretrovirinae* member they tested, specifically BLV. Despite the lack of identifying Pol III transcribed miRNAs from the limited set of other retroviruses examined, it is likely that additional RNA viruses will be found to encode Pol III transcribed miRNAs due to the evolutionary conservation of this mechanism. Furthermore, the ability of an RNA virus to produce miRNAs from a Pol III transcript, as demonstrated in this paper, provides a mechanism by which RNA viruses can produce miRNAs while avoiding any detrimental effects on its own transcribed genome, such as cleavage by the Drosha/DGCR8 Microprocessor complex.

## Discussion

The work done by Kincaid *et al.* is a welcome discovery that should alter a common misconception that RNA viruses cannot encode miRNAs. In light of these findings, we wish to discuss a general set of rules regarding the presence of viral miRNA in infected cells. They include:

1. Viral miRNAs or other functional sncRNAs must be detectable at least by deep sequencing methods and mediate a biological effect. Importantly, these findings must be experimentally verified by multiple methods and multiple labs.

2. The function of the viral miRNA must be described and conserved.

3. The miRNA sequence must be conserved across clades.

**Figure 1:**
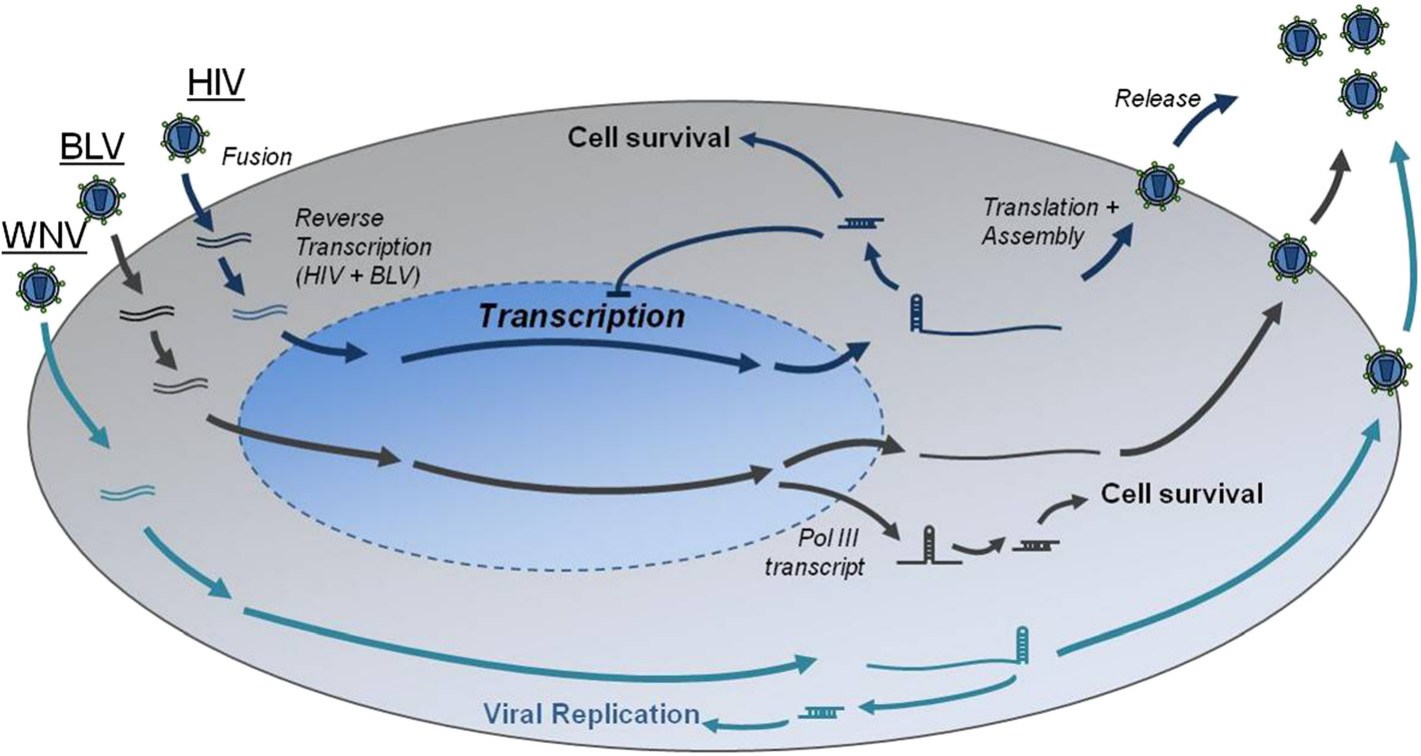
**Expression and biological function of miRNAs encoded by three RNA viruses.** Two retroviruses and one flavivirus, HIV, BLV, and WNV, respectively, have been shown to encode functional miRNAs within their genomes. The identified viral miRNAs have been shown to elicit varied responses including increased cell survival and viral replication, as well as inhibiting viral transcriptional, thereby inducing latency. Additionally, the biosynthesis of the miRNAs from each virus has been shown to occur through diverse mechanisms of action, each of which diverges from the canonical host miRNA maturation process.

Taking these three points into consideration, we propose that BLV derived miRNAs are actually the third instance of an RNA virus that expresses miRNAs. The other two examples are HIV-1 and West Nile Virus (WNV), of which HIV-1 has been studied to the greatest extent (Figure 1). Furthermore, these general rules should be examined when testing the existence of all future viral miRNAs.

To the first point for HIV-1, in 2004 Bennasser *et al.* reported on potential HIV-1 encoded miRNA candidates and their likely cellular targets using computer algorithms [4]. Then in 2007, our lab first verified that HIV-1 contained miRNAs by using a number of *in vitro* assays [5] and later by cloning of the mature miRNAs derived from both the 5^′^ and 3^′^ end of the TAR structure [6]. Since that time these findings have been confirmed by several independent labs. For instance, Berkhout and colleagues utilized state of the art SOLiD deep sequencing technology to analyze over 16 million sequences including 5 million small RNA sequences of which 2.6 × 10^4^ were HIV-1 specific sncRNAs [7]. They identified sncRNAs from the TAR element as the most prominent and many anti-sense sequences from the negative strand that could function as siRNAs. Previous pyrosequencing of HIV-1 sncRNAs by the Jeang lab also showed TAR derived miRNAs to be the most prevalent with a copy number of 3 × 10^3^ copies per cell, which is comparable to the highest expressing host miRNAs [8]. While the specific copy number of the individual BLV derived miRNAs was not given, counts for each BLV miRNA sequence of up to 4.9 × 10^4^ exceeded the maximum 3.6 × 10^4^ counts of the highest expressing host cell miRNA [2]. These relatively high miRNA counts of both the BLV and HIV-1 encoded miRNAs indicate the biological relevance of each. More recently, Althaus *et al.* developed and employed an enrichment strategy that isolated low abundance HIV-1 derived sncRNAs that boosted their proportion in the cloned pool by over 100-fold [9]. The sncRNA sequences derived from this enrichment process included matches to three previously identified HIV-1 miRNAs including hiv1-miR-N367, hiv1-miR-TAR-3p, and hiv1-miR-H1. Furthermore, Wagschal *et al.* elucidated the novel molecular mechanism by which the abortive TAR transcripts are released and processed into functional sncRNAs from promoter-proximal paused RNA Polymerase II (Pol II) [10]. The findings from these aforementioned studies supplants earlier work that failed to identify miRNAs from retroviruses [11]. The primary improvement with these more recent sequencing studies that positively identified HIV-1 derived sncRNAs is the depth of clones sequenced or enrichment of target clones, which leads to a greater statistical power to detect low abundance sncRNAs.

Using a similar methodology, the miRNA derived from the 3′ stem-loop structure of the WNV genomic RNA was first identified using bioinformatic techniques followed by Northern blot detection and small RNA sequencing from infected Aag2 mosquito cells [12]. Furthermore, it was found that the processing of the WNV pre-miRNAs into the mature 21nt miRNAs was Dicer1 dependent. As this WNV miRNA has currently only been detected from one strain of the virus and only from the vector Aag2 mosquito cell line, further validation of this viral miRNA is required. However, the ability of WNV to encode a miRNA increases the likelihood that other related cytoplasmically replicating RNA viruses encode their own miRNAs.

As to point 2, the common functions of viral miRNAs are to control viral life cycle, cell survival and immune evasion, of which the BLV, WNV, and HIV-1 miRNAs can effectively impact. Specifically, one of the BLV miRNAs studied in more detail was found to be a mimic of the oncogenic host miR-29 [2]. In regards to the WNV miRNA, it was shown to up-regulate the host GATA4 expression, which increased viral genomic RNA replication [12]. Lastly, for the HIV-1 viral miRNAs, the TAR-derived miRNA has the ability to induce latency, which will be discussed in detail below.

Furthermore, in examining point 3, the HIV-1 TAR sequence is highly conserved at the seed sequences with 95% identity across nearly all HIV-1 isolates. Likewise with the miRNAs identified from BLV, four of the five predicted seed sequences were identical across the seven isolates examined [2]. As previously mentioned, the WNV encoded miRNA will need to be validated by checking seed sequence homology across other related clades.

In further exploring point 2, we offer an explanation of one specific viral miRNA being effective in the nucleus which may directly contribute to viral latency. Specifically, high abundance unprocessed miRNAs, such as TAR, and increased expression of the processed 5^′^ or 3^′^ terminus of the stem- and loop structure [10,13], results in transcriptional gene silencing (TGS) of the HIV-1 promoter. TGS events can be easily scored *in vivo* by using ChIP assays, as cells which have increased levels of TAR miRNA show recruitment of enzymes (i.e., HDACs and methyltransferases) involved in local epigenetic modifications and transcriptional memory on the HIV-1 and specific cellular promoters [10,13]. This mode of 100% small RNA-DNA promoter complimentarity complex or small RNA-mRNA hybridized complex may not only explain the TGS seen on the HIV-1 promoter, but also thousands of cellular promoters that have short RNAs resulting from an stalled, unprocessive Pol II bound to their promoters. The recent work by Wagschal *et al.* specifically addressed this possibility and found that TAR derived sncRNAs indeed inhibited HIV-1 LTR activity and induced chromatin remodeling. Moreover, they found comparable Microprocessor recruitment and chromatin remodeling at human endogenous retroviral sites within the genome indicating a more ancient conserved mechanism by which retroviruses can be silenced [10].

Furthermore, the assumption that RNA viruses that encode a miRNA may have their genome degraded by Drosha, rendering them effete, may not be correct. Along these lines, Berkhout’s lab has published studies demonstrating that viral genomic RNA is shielded from RNase activity [14]. Additionally, a recent paper examined the expression of cellular miRNAs inserted into the Nef locus of an infectious HIV-1 genome [15]. In this work it was shown that while insertion of miRNAs that are efficiently processed did decrease viral replication, insertion of less efficiently matured miRNAs did not alter replication. Moreover, the non-canonical miRNA biosynthesis pathways that have been recently identified for the generation of the BLV miRNAs and TAR derived miRNAs effectively evade potential full-length viral transcript cleavage and degradation [2,10].

## Conclusion

Taken together the work by Kincaid *et al.* describes a fascinating new finding on yet another retrovirus that not only contains a viral miRNA made through a robust Pol III promoter but also the possibility of control of viral and cellular genes that could potentially make the host more susceptible to viral regulation. Perhaps this line of research is exactly what is needed to truly uncover the mysteries of retroviral regulation of multiple genes through an RNA element in higher eukaryotic cells.

## Competing interests

The authors declare that they have no competing interests.

## Authors’ contributions

ZK, GCS, and FK wrote the manuscript. All authors read and approved the final manuscript.

## References

[B1] Liang D Lin X Lan K Looking at Kaposi’s Sarcoma-Associated Herpesvirus-Host Interactions from a microRNA Viewpoint Front Microbiol 2011 2 271 22275910 10.3389/fmicb.2011.00271 PMC3258008

[B2] Kincaid RP Burke JM Sullivan CS RNA virus microRNA that mimics a B-cell oncomiR Proc Natl Acad Sci U S A 2012 109 3077 3082 10.1073/pnas.1116107109 22308400 PMC3286953

[B3] Pfeffer S Sewer A Lagos-Quintana M Sheridan R Sander C Grasser FA van Dyk LF Ho CK Shuman S Chien M Identification of microRNAs of the herpesvirus family Nat Methods 2005 2 269 276 10.1038/nmeth746 15782219

[B4] Bennasser Y Le SY Yeung ML Jeang KT HIV-1 encoded candidate micro-RNAs and their cellular targets Retrovirology 2004 1 43 10.1186/1742-4690-1-43 15601472 PMC544590

[B5] Klase Z Kale P Winograd R Gupta MV Heydarian M Berro R McCaffrey T Kashanchi F HIV-1 TAR element is processed by Dicer to yield a viral micro-RNA involved in chromatin remodeling of the viral LTR BMC Mol Biol 2007 8 63 10.1186/1471-2199-8-63 17663774 PMC1955452

[B6] Klase Z Winograd R Davis J Carpio L Hildreth R Heydarian M Fu S McCaffrey T Meiri E Ayash-Rashkovsky M HIV-1 TAR miRNA protects against apoptosis by altering cellular gene expression Retrovirology 2009 6 18 10.1186/1742-4690-6-18 19220914 PMC2654423

[B7] Schopman NC Willemsen M Liu YP Bradley T van Kampen A Baas F Berkhout B Haasnoot J Deep sequencing of virus-infected cells reveals HIV-encoded small RNAs Nucleic Acids Res 2012 40 414 427 10.1093/nar/gkr719 21911362 PMC3245934

[B8] Yeung ML Bennasser Y Watashi K Le SY Houzet L Jeang KT Pyrosequencing of small non-coding RNAs in HIV-1 infected cells: evidence for the processing of a viral-cellular double-stranded RNA hybrid Nucleic Acids Res 2009 37 6575 6586 10.1093/nar/gkp707 19729508 PMC2770672

[B9] Althaus CF Vongrad V Niederost B Joos B Di Giallonardo F Rieder P Pavlovic J Trkola A Gunthard HF Metzner KJ Fischer M Tailored enrichment strategy detects low abundant small noncoding RNAs in HIV-1 infected cells Retrovirology 2012 9 27 10.1186/1742-4690-9-27 22458358 PMC3341194

[B10] Wagschal A Rousset E Basavarajaiah P Contreras X Harwig A Laurent-Chabalier S Nakamura M Chen X Zhang K Meziane O Microprocessor, Setx, Xrn2, and Rrp6 co-operate to induce premature termination of transcription by RNAPII Cell 2012 150 1147 1157 10.1016/j.cell.2012.08.004 22980978 PMC3595997

[B11] Lin J Cullen BR Analysis of the interaction of primate retroviruses with the human RNA interference machinery J Virol 2007 81 12218 12226 10.1128/JVI.01390-07 17855543 PMC2169020

[B12] Hussain M Torres S Schnettler E Funk A Grundhoff A Pijlman GP Khromykh AA Asgari S West Nile virus encodes a microRNA-like small RNA in the 3' untranslated region which up-regulates GATA4 mRNA and facilitates virus replication in mosquito cells Nucleic Acids Res 2012 40 2210 2223 10.1093/nar/gkr848 22080551 PMC3300009

[B13] Carpio L Klase Z Coley W Guendel I Choi S Van Duyne R Narayanan A Kehn-Hall K Meijer L Kashanchi F microRNA machinery is an integral component of drug-induced transcription inhibition in HIV-1 infection J RNAi Gene Silencing 2010 6 386 400 20628499 PMC2902143

[B14] Westerhout EM ter Brake O Berkhout B The virion-associated incoming HIV-1 RNA genome is not targeted by RNA interference Retrovirology 2006 3 57 10.1186/1742-4690-3-57 16948865 PMC1569866

[B15] Klase Z Houzet L Jeang KT Replication competent HIV-1 viruses that express intragenomic microRNA reveal discrete RNA-interference mechanisms that affect viral replication Cell Biosci 2011 1 38 10.1186/2045-3701-1-38 22112720 PMC3256098

